# Implementing functional electrical stimulation clinical practice guidelines to support mobility: A stakeholder consultation

**DOI:** 10.3389/fresc.2023.1062356

**Published:** 2023-01-26

**Authors:** Lisa Brown, Tamsyn Street, Adine Adonis, Therese E. Johnston, Simona Ferrante, Jane H. Burridge, Catherine Bulley

**Affiliations:** ^1^ Department of Physical Therapy, Boston University, Boston, MA, United States; ^2^ Salisbury NHS Foundation Trust, Salisbury, United Kingdom; ^3^ Department of Physiotherapy, Imperial College Healthcare NHS, London, United Kingdom; ^4^ Department of Physical Therapy, Arcadia University, Glenside, PA, United States; ^5^ Department of Electronics Information and Bioengineering, Politecnico di Milano, Milan, Italy; ^6^ University of Southampton, Southampton, United Kingdom; ^7^ Queen Margaret University, Musselburgh, United Kingdom

**Keywords:** clinical practice guidelines, functional electrical stimulation, upper motor neuron conditions, neurorehabilitation, rehabilitation

## Abstract

Functional Electrical Stimulation (FES) has been used to support mobility for people with upper motor neuron conditions such as stroke and multiple sclerosis for over 25 years. Recent development and publication of clinical practice guidelines (CPGs) provide evidence to guide clinical decision making for application of FES to improve mobility. Understanding key barriers to the implementation of these CPGs is a critical initial step necessary to create tailored knowledge translation strategies. A public involvement and engagement consultation was conducted with international stakeholders including researchers, clinicians and engineers working with FES to inform implementation strategies for CPG use internationally. Reflexive thematic analysis of the consultation transcripts revealed themes including inconsistent use of CPGs, barriers to implementation such as limited access to FES and low clinician confidence, and the need for a tiered education approach with ongoing support. Insights derived from this consultation will inform the development of knowledge translation strategies to support the next steps to implementing FES use for mobility.

## Introduction

Functional electrical stimulation (FES) to improve mobility involves electrical stimulation of peripheral nerves in the lower extremity for improving locomotion or strengthening muscles (1). Clinical guidelines that recommend FES to improve foot drop in people with upper motor neuron conditions have been in existence since 2009, since the introduction of National Institute for Health and Care Excellence (NICE) guidance (2). Clinical practice guidelines (CPGs) are defined by the Institute of Medicine (IOM) as “a set of recommendations, involving both the evidence and value judgments regarding benefits and harms of alternative care options, addressing how patients with that condition should be managed, everything else being equal” (3). CPGs supporting healthcare professionals to improve lower limb function and mobility in individuals with stroke and upper motor neuron dysfunction are relatively new (4) or in development (5). These CPGs provide a synthesis of current scientific evidence, expert clinical experience, and patient preferences. CPGs have the potential to support clinical decision making, reduce practice variability, assist in educating patients and caregivers on best practices, aid policy makers in the allocation of healthcare resources, and inform the development of educational courses (3). However, the benefits to patients through implementation of evidence into practice is often not realised, with only about 14% of published evidence making an impact on clinical practice after an average of 17 years (6). Insufficient clinical impact achieved indicates that a more active approach is required through barrier assessment and tailored knowledge translation implementation strategies (3, 4).

Knowledge translation is defined by the National Center for the Dissemination of Disability Research (NCDDR) as “The collaborative and systematic review, assessment, identification, aggregation, and practical application of high-quality disability and rehabilitation research by key stakeholders (i.e., consumers, researchers, practitioners, and policymakers) for the purpose of improving the lives of individuals with disabilities” (5). Increasing clinical implementation through knowledge translation must be a dynamic and iterative process to effectively impact the health and wellness of individuals and to strengthen the healthcare system (7). The Knowledge to Action Framework developed by Graham et al. suggests that once knowledge is created, it must be put into action through a series of dynamic phases that include assessing barriers and facilitators to knowledge use, developing implementation interventions, and adapting strategies to specific local needs (7). The CPG development process relating to FES use for improved mobility has synthesized evidence of different types to create new knowledge. The subsequent goal of implementing FES CPGs is to support effective and efficient clinical decision making, enabling the best possible care and thereby improved patient outcomes.

The next step in this knowledge to action plan includes the assessment of barriers to knowledge use to ensure that the CPGs achieve positive change. To achieve this goal, it imperative to gain the thoughts and perspectives of clinicians and other stakeholders using or considering the use of FES (8). Public engagement and involvement consultations play an important role in the dissemination of research and can improve the quality, relevance, and ultimately the usefulness of the knowledge to action products (9). Publications on this topic by Howlett et al., 2018, Auchstaetter et al. 2016, and Tedesco Triccas et al. 2021 identified barriers to FES use including gaps of education or training for FES use and lack of resources (10–12). Each of these surveys were completed prior to the publication of the recent evidenced based CPG in 2021, and were online surveys only distributed to one specific region, potentially limiting the global application of the results. Prior publications also did not include interactive discussion which is a critical element to understand the people's views and lived experiences (13).

While barriers have been previously documented, the ultimate focus of this consultation was to understand how to move beyond all these barriers. Thus, the purpose of this public engagement and involvement consultation was to engage in discussion with individuals from a variety of countries that are using FES to consider current practice patterns, use of CPGs, perceived barriers to CPG and FES use, and to gain an understanding of priorities for education and training. This international perspective will be used to inform the design of international CPG implementation strategies including education outreach that will support FES use to improve mobility.

## Methods

A public engagement and involvement consultation was conducted to obtain the viewpoints of key stakeholders involved in the provision of FES clinical services to patients (14). Three virtual workshops were held between September 2021 and May 2022, with a combined total of 172 participants. Information was compiled to address the purpose of the consultation, which was to gather information from individuals using FES from a variety of countries to document current practice patterns, perceived barriers to FES use, and use of CPGs. This international perspective was sought to assess the barriers to knowledge use to identify, design and implement educational needs across different geographical areas and health systems.

### Consultation development

A discussion plan for the consultation was developed by the authors of the recent Clinical Practice Guideline (TJ, LB) (4) and by a CPG in development (TS, CB, AA, SJ, JB) (15). The expert author panel included academics, researchers and clinicians with experience using FES. The intention of the discussion plan was to use a pragmatic approach to develop a brief series of guided open-ended questions and follow-up questions, (Table 1). The aim of the consultation was to use a responsive interviewing structure to provide participants an opportunity to describe their experiences using FES in the real world, and to engage in the discussion about the role of CPGs and the potential next steps of implementation (13). The discussion plan covered topics related to participant role and interest in FES, geographical location, practice patterns with FES, knowledge of and barriers to use of CPGs and perceived educational or training priorities for translation of evidence into clinical practice.

**Table 1:** Discussion plan.

**What are your roles/interests relating to Functional Electrical Stimulation (FES)?**
**What type of FES are you familiar with**?
**Is FES used clinically in your region/country?**
If you do not use FES, can you share your reasons with us?
**Does your region/country use any FES clinical guidelines currently? Which? How?**
If you do not use clinical guidelines, can you share your reasons with us?
Is there anything that you think will make it more likely that clinical guidelines will be used in your region/country?
Do you think there would be/are any barriers to using/implementing clinical guidelines relating to FES for mobility? What may they be?
**In what way do you use FES in your clinical practice?**
If you use FES, how do you decide which patients are able to benefit?
**Do you feel that that there is a need to use guidelines differently in different countries?**
If so, what would these needs be?
**What are the priorities in this area for development, education, and training?**

### Workshop and consultation administration

All consultations were held virtually using the videoconferencing platform Zoom (Zoom Video Communications, 2016) (14). The first session was held as part of a workshop entitled “Development of Clinical Guidelines for FES in Mobility” during the international virtual Rehab Week 2021 conference. This well-established biennial conference is sponsored in collaboration with the International Functional Electrical Stimulation Society (IFESS) among other societies and typically attracts a variety of participants including researchers, clinicians, engineers, and industry specializing in FES. The workshop was advertised through RehabWeek conference promotions and through social media channels. The workshop was open to all conference attendees, and anyone who was present for the initial introductory portion of the workshop was invited to participate in the consultations portion. Participants were provided with the option to participate in the workshop without participating in the consultation. The second consultation occurred during a virtual international workshop titled “Bridging the Gap between Functional Electrical Stimulation Research and Clinical Implementation” sponsored by the International Functional Electrical Stimulation Society (IFESS) and the Association of Chartered Physiotherapists In Neurology (ACPIN). This freely available workshop was made available through IFESS and ACPIN email distribution lists and social media channels. This virtual workshop included an initial introduction followed by an invitation to participate in a voluntary small group consultation. The third consultation included invited clinicians from the United States. Stakeholders working in the area of FES were invited through email requests from the panel. This consultation started with an introduction to the project after which attendees were invited to participate in the small-group consultations. All consultations were voluntary, and participants were not compensated for their time. Consultations were offered on different days and times to accommodate the varied time zones of the expert panel and the participants. All consultations were offered virtually due to ongoing Covid 19 pandemic restrictions, and to encourage and accommodate a broader audience.

The beginning of each workshop aimed to provide background information about the development and implementation of CPGs for FES. Participants were given the opportunity to ask questions prior to participation in the optional consultation session. Participants were informed of the intended use of their views during the consultation, and that their views would be recorded. and provided with the opportunity to opt-out. Joining the optional zoom breakout room indicated agreement to participate in the consultation. Follow-up consultation was then held in zoom break-out rooms to gain insights into current practice patterns and key challenges with FES and CPG implementation.

### Consultation outcomes

Audio-recordings of the consultations were recorded in zoom and transcribed verbatim. Transcripts were compared to the audio recordings by expert panel members for accuracy and all participant information was deidentified to maintain anonymity. The transcripts were reviewed using an iterative process, and themes were identified and coded using NVivo Qualitative Analysis software; QSR International, Burlington, MA. Using a framework analysis two members of the expert panel (LB, TS) read the transcripts from the open-ended questions to familiarize themselves with the responses (13, 16). Using reflexive thematic analysis with an iterative process, the transcripts were read again by each reviewer and initial codes were identified (13, 17). The 2 reviewers (LB, TS) then discussed and compared initial codes and categorized similar conceptual codes into emerging themes related to each question. Themes were agreed upon and organized by each objective of the consultation including participant demographics and practice patterns with FES, awareness of CPGs, and perceived barriers to FES use (17).

## Results

### Background information and practice patterns

The virtual consultation was provided on 3 different occasions. Of the 172 participants across all 3 workshops, a total of 18 chose to fully participate in the consultation. Geographical representation was oriented around Europe and North America and included representation from Canada, Denmark, Ireland, Italy, the Netherlands, the United Kingdom, and the United States. Participants were predominantly physiotherapists with representation from clinicians, educators, researchers, and engineers (Table 2). Participants were asked which diagnoses they considered using FES as an intervention. The most common diagnoses included stroke, spinal cord injury, multiple sclerosis, brain injury, transverse myelitis, and cerebral palsy.

**Table 2:** Demographic information.

Occupation	% of participants
Physiotherapist	72
Orthotist	<1
Engineer	11
Researcher	11
**Geographic location**	
United Kingdom	44
United States	22
Canada	11
Netherlands	<1
Italy	<1
Ireland	<1
Unknown	<1

Participants’ experience levels with FES ranged from the novice to expert level. Interventions with FES included to improve patient mobility (dropped foot) or using FES as a therapeutic modality within intervention sessions for functional training or focal muscle strengthening. Some participants reported using devices such as FES cycling to enhance exercise participation. Frequencies of use of FES in clinical practice varied from sporadic to daily. Most clinic settings were described as providing a broad range of interventions, while some described the clinical setting in which they work as a FES specialty centre or service to which individuals are referred for the primary purpose of assessment for and interventions using FES. Participants with the highest confidence relating to FES and most consistent use noted having access to a training structure and support network.

### Current use of CPGs

**Figure 1:**
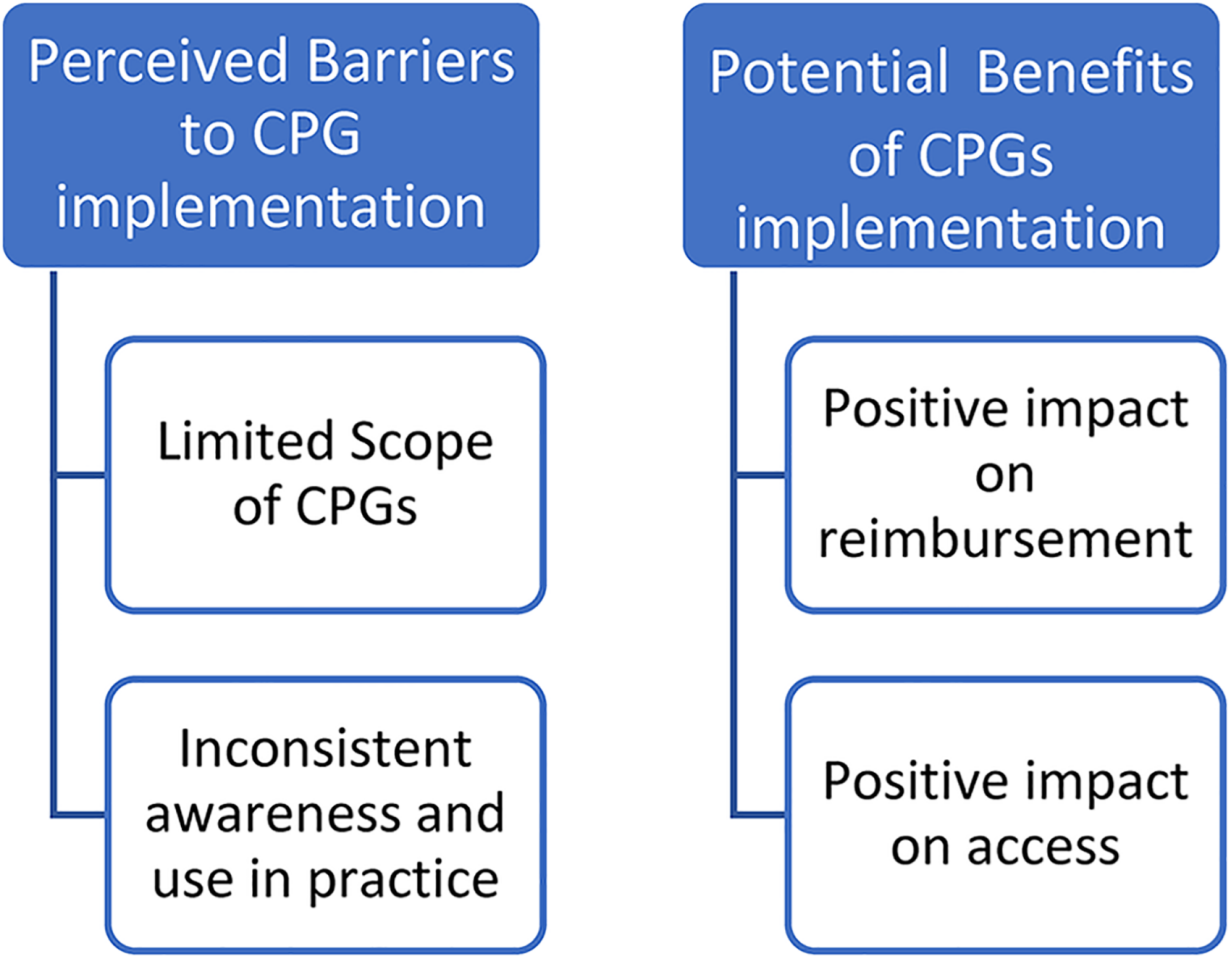
CPG use and implementation. CPG, clinical practice guideline.

Participants were asked to discuss their current use of clinical guidelines in practice and any barriers that may impact FES use or the implementation of a CPG. Participants were aware of the NICE guidelines published in 2009 but were inconsistently aware of clinical guidelines available for use of FES post stroke recently published in 2021 (2, 4). When discussing barriers to implementation of FES CPGs themes included limitations in the scope of CPGs and inconsistent awareness and use in practice. When discussing CPG scope, one participant with an academic background noted: “*I do not believe we are asking the right clinical questions before we go into those guidelines. What specifically we are missing is: what does the patient want to gain out of using the technology?*” Participants commented that current guidelines needed to be more specific to health conditions or interventions and did not appear to clearly define a clinical decision-making process. For example, a practitioner said: “*to actually use them for the clinical practice, or within, they are not descriptive, or descriptive enough to, they don't tell you how to do it, just that there is evidence out there, it has a benefit*.” Participants also felt that a CPG may not provide enough detail or may be difficult to carry over into facility guidelines: “*we've had discussions about the FES and AFO clinical practice guidelines …. but we don't have hospital or a department guideline for clinical, so we don't have those for anything*.” Potential benefits to CPGs were also noted by participants and included a potential to positively impact reimbursement and access. “*I think in the UK the guidelines help with funding … and with a guideline at least there is a legitimate background for ordering it*.” (Figure 1)

### Perceived barriers to FES use

**Figure 2:**
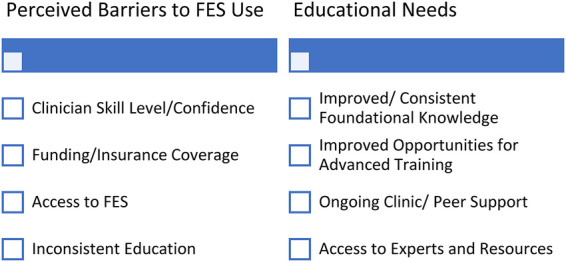
Barriers to FES use and educational needs. FES, functional electrical stimulation.

Themes related to barriers to FES use included clinician skill level and confidence, limitations in funding, and inconsistent educational offerings. Clinician skill level and confidence with FES technology were mentioned by several participants. One clinician stated: “*I trained up on quite a few different devices but have not had access to those devices, and I think my biggest one is noticing how that lack of confidence just writes you off so quickly*”. Other themes included economic barriers such as limited insurance coverage and constraints accessing FES within a given geographical location. Clinicians in the United Kingdom described unequal provision of healthcare resources depending on the person's geographic area: “*It's a post code lottery; it depends on what area of the country you live in*”. Many participants across geographic regions noted various challenges related to timely access: “*I think a lot of people are aware of it and know the benefits but accessing it is something, it's launching such an administrative journey to try and get that it doesn't result in success*.” … “*I did qualify in using FES but it was very difficult to access in my clinical practice, so I reverted to orthotic practice as standard*.” (Figure 2).

Participants described varied and inconsistent educational offerings from entry level to advanced practice. Some participants noted FES was presented in pre-registration neurological modules, while others commented that it may not be introduced at the entry level at all: “*It's mixed, it depends on the university…. I do think there is definite differences in the education, who learns about these and who does not*.” Some participants attended courses on FES application as practicing clinicians. However, a lack of support following this instruction was noted as a barrier to consistent implementation: “*personally, my experience was I went on the course, I got to understand it, I did quite a bit on the course, but then I came locally, and I saw a few patients, and when I was only seeing one, two a month, you don't build up the expertise and in the end I thought that I’m not getting enough practice to maintain my skills*”.

### Identification of educational needs to inform implementation strategies

During the consultations participants were asked to discuss strategies that would impact their likelihood of including FES and CPGs in clinical practice, the priorities for development, education, and training, and if there is a need to use guidelines differently in different countries. Overall themes for educational needs consisted of improved foundational knowledge, ongoing clinical and peer support, and access to an expert or consistent resource. Participants identified a need for improved foundational knowledge of CPGs and FES applications at the entry level of education, and the need for accredited basic and advanced training courses beyond entry level education. One participant stated: “*it really isn't something you can teach them in a day clinic, a day's course or a two-day course and then let them walk away without giving them support*.”

Ongoing clinical/peer support, “hands-on” problem solving, and regular updates were suggested by most participants as strategies to improve implementation. Participants that worked in settings with successful and sustained FES use described a tiered approach to clinician training that included education aligned with mentored practice opportunities. One person explained: “*I set up a staged programme, they learn about technology, they learn about its functions, they learn how to manipulate it etc., they learn about the theory behind it, they then get to practice it on themselves, then they shadow in clinics to watch it being put on other people. Then by the fourth – fifth week they are starting to actually apply it themselves, on patients while they have got someone else in the room that's shadowing them. Then by the sixth, seventh, eight week they are left to practice on their own with a support mechanism around them where they can ask any questions*.” Finally, appointing a trained and dedicated expert as a resource in a clinic was viewed as an effective strategy to enhance FES use and clinician confidence: “*I think role modelling from other colleagues helps*.” (Figure 2).

When discussing whether CPGs need to be individualized in different countries, participants did not believe that each country or region required a unique set of guidelines. One participant commented that “*any of the CPGs that are already developed can be given to any other country or part of the world*”. Some participants noted modifications such as accurate translation to different languages and considerations for cultural adaptations should be considered.

## Discussion

The aim of these consultations was to gather preliminary information from individuals using FES to understand current FES practice patterns, including use of CPGs and the perceived barriers to FES use and to gain an understanding of priorities for education and training. According to Grimshaw et al. (2012), “*planned knowledge translation for healthcare professionals and consumers is more likely to be successful if the choice of knowledge translation strategy is informed by an assessment of the likely barriers and facilitators*” (8). Therefore, the insights gained from these conversations will be used to inform international CPG knowledge translation strategies including the educational needs for FES use to improve mobility.

The consultations included participants who ranged from the novice to expert level, and the applications discussed included functional retraining, muscle strengthening and exercise enhancement across varied neurologic diagnoses. The diversity in the backgrounds of participants of these small groups provides a wide range of insights into the current practice and barriers related to FES use, which may better inform potential educational needs.

Multiple barriers to effective implementation of FES were documented including inconsistent access to FES devices, decreased awareness of the evidence supporting FES use, and variability of FES education and supports contributing to a lack of confidence and overall use of FES. These barriers are similar to those identified in current research on barriers in FES use, which highlights the need for improved educational and implementation strategies with considerations for behaviour change (6, 10, 11, 18). The behaviour change research documents a lack of access or awareness of current research and a lack of clinician efficacy interpreting the research as barriers to evidence-based practice (19). The similarities in barriers identified across geographic areas suggests the potential for a global approach to implementation of CPGs for FES could be effective. Importantly, this strategy would still need to include local stakeholder involvement for individual regions in the intervention process (20).

It is important to understand the perceived knowledge gaps, including where and how they occur, when considering education strategies as a component of the knowledge to action plan. The inconsistent introduction of CPGs and FES during entry level physiotherapy education was noted as a barrier in the group discussions, indicating that there is a need for the development of standardized and consistent introduction to CPGs and FES at the entry level. The need for advanced training courses beyond the entry level was also vocalized, indicating implementation strategies designed for postgraduate accredited or competency based continuing professional development courses are needed that cater to varying levels of clinician expertise.

For successful translation of CPGs on FES into clinical practice, development of multimodal knowledge translation strategies is required to improve practice and change clinician behaviour (18–20). A systematic review by Berube et al. (2018) provides guidance that may assist in the implementation of CPGs related to FES (10). Successful increases in physiotherapists’ knowledge and awareness of musculoskeletal guidelines were achieved using a variety of techniques, such as professional educational materials, presentations, and marketing materials. More positive patient outcomes were seen with face-to-face continuing education courses that included practical application as compared to passive learning from reading documents (21). Implementation interventions that were multifaceted and extended beyond a brief time period were found to be more successful, with one study suggesting that up to 8 days of training, followed by monitoring, are needed for behavioural change (22). This systematic review concluded that implementation interventions must be of sufficient length, use practical application tools, and allow time for questions and feedback (21).

Efforts to improve clinical decision making may be further supported by the recent development of a decision-making tool. The FES Clinical Decision-Making Tool was developed and tested its content validity with Canadian physical and occupational therapists (23). The tool seeks to facilitate clinical decision making with regards to appropriate parameters to use during FES treatment which is an area not currently well represented in available clinical guidelines. The FES clinical decision-making tool has not been validated in clinical practice yet but can be considered as a component of a knowledge to action plan.

Participants who were at the expert level of practice with FES attributed success in practice to a strong support network. This finding is consistent with the knowledge translation literature that suggests establishing a local champion or knowledge broker who is responsible for supporting ongoing discussions, interactive educations, and clinical consultations as a critical step in the knowledge to action process (7, 19). Other strategies should focus on organizational, community, system, cultural, and policy levels, aiming to enhance motivation, resources, and organizational dynamics (24, 25).

In conclusion, the preliminary consultation assisted in the understanding of global barriers to CPG and FES use to inform next steps in the knowledge-to-action process to support implementation methods. It is important that evaluation frameworks are used to seek feedback on the implementation strategies and behaviour change techniques to enable evaluation of the success of CPG implementation (25, 26). The information collected can be used to improve the effectiveness knowledge translation strategies and to provide guidance about further research needed to improve CPGs. New research should then be reviewed and integrated into the CPG on a regular basis. A dynamic implementation approach will promote relevance and usefulness of CPGs, closing the gap between research and clinical practice.

## Limitations

While individuals with varied backgrounds were invited to participate and the consultations sessions were held at different times to accommodate various time zones, many of the participants were physiotherapists with representation from Europe, Canada, and the United States, which limits the perspectives that provided input in this consultation. The number of participants in this initial consultation is small limiting generalizability.

## Data Availability

The raw data supporting the conclusions of this article will be made available by the authors, without undue reservation.
